# Identifying source populations for the reintroduction of the Eurasian beaver, *Castor fiber* L. 1758, into Britain: evidence from ancient DNA

**DOI:** 10.1038/s41598-018-21173-8

**Published:** 2018-02-09

**Authors:** Melissa M. Marr, Selina Brace, Danielle C. Schreve, Ian Barnes

**Affiliations:** 1 Department of Geography, Royal Holloway University of London, Egham Hill, Egham, Surrey, TW20 0EX UK; 2 Department of Earth Sciences, Natural History Museum London, Cromwell Road, South Kensington, London, SW7 5BD UK

## Abstract

Establishing true phylogenetic relationships between populations is a critical consideration when sourcing individuals for translocation. This presents huge difficulties with threatened and endangered species that have become extirpated from large areas of their former range. We utilise ancient DNA (aDNA) to reconstruct the phylogenetic relationships of a keystone species which has become extinct in Britain, the Eurasian beaver *Castor fiber*. We sequenced seventeen 492 bp partial tRNAPro and control region sequences from Late Pleistocene and Holocene age beavers and included these in network, demographic and genealogy analyses. The mode of postglacial population expansion from refugia was investigated by employing tests of neutrality and a pairwise mismatch distribution analysis. We found evidence of a pre-Late Glacial Maximum ancestor for the Western *C. fiber* clade which experienced a rapid demographic expansion during the terminal Pleistocene to early Holocene period. Ancient British beavers were found to originate from the Western phylogroup but showed no phylogenetic affinity to any one modern relict population over another. Instead, we find that they formed part of a large, continuous, pan-Western European clade that harbored little internal substructure. Our study highlights the utility of aDNA in reconstructing population histories of extirpated species which has real-world implications for conservation planning.

## Introduction

Reintroduction programs are an essential tool in the restoration of viable, free-ranging populations^1^ but over 50 percent result in failure^2^. Identification of suitable source population/s from which to select founding members for the restoration area is a critical factor in increasing the likelihood of success^1,3,4^. Structured differences in the distribution of phenotypic traits and adaptive genetic variation arise between populations as the result of divergent evolutionary processes and adaptation to local conditions^5,6^. These variations in genetic architecture create differential responses to novel environments, stressors and pathogens and create variation in the reintroduction potential of populations^4^. Assessment of genetic parameters across source population/s is, therefore, now a chief objective in translocation planning^3^.

**Figure 1:**
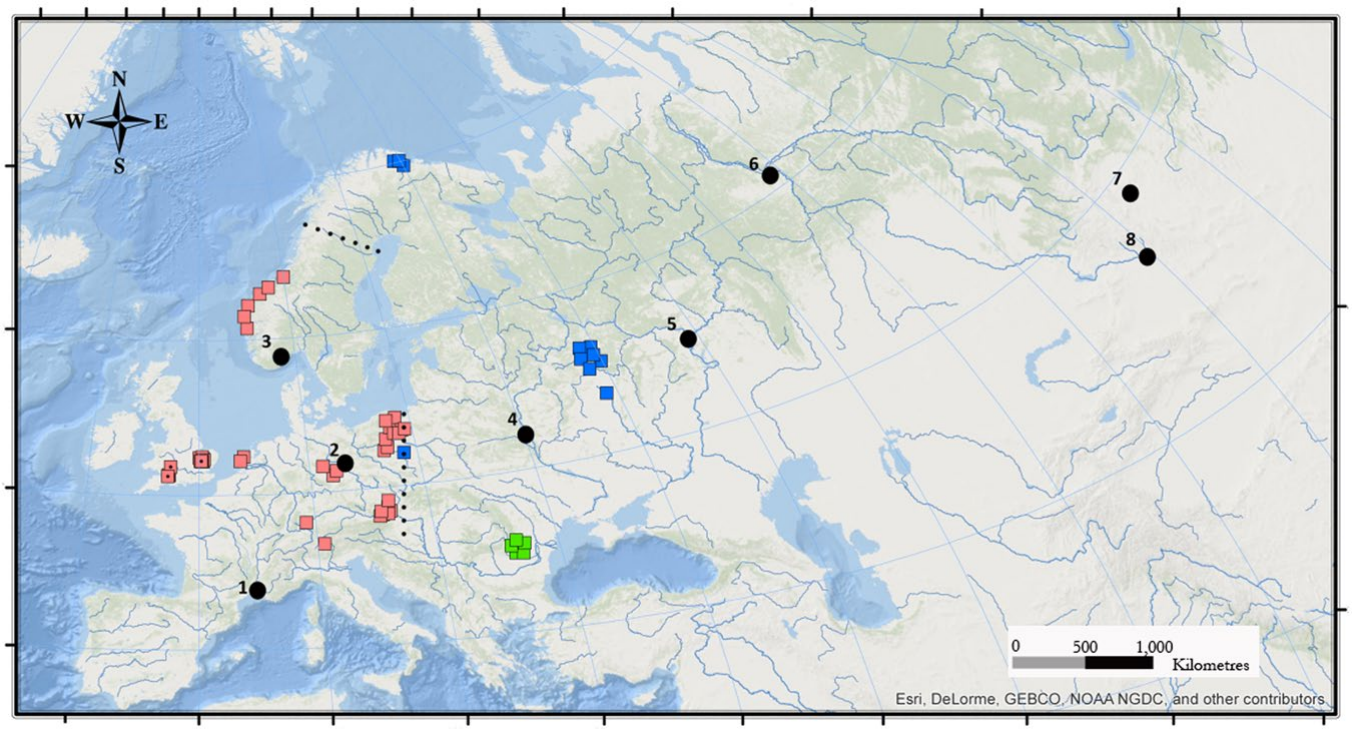
Map of sampling localities for ancient *Castor fiber* individuals. The Western clade (pink) and Eastern clade (blue) have two Holocene contact zones; one in Eastern Europe and one in NW Scandinavia. A now extinct clade existed in the Danube Basin (green) until the late Holocene^26^. Sampling localities for the British data are indicated with dotted square icons, all other points refer to those generated by Horn *et al*.^26^. Only localities from where successful sequences were retrieved are shown. These represented 17 specimens from the SW and SE of England (SI Table 2). Approximate locations of extant relict populations (1 to 8) from where modern *C. fiber* haplotypes derive are shown in black. *Map created using ArcMap v10.5*, *accessed at* www.esri.com. *Data was sourced from Natural Earth Data*, www.naturalearthdata.com.

The beaver, *Castor fiber*, is the largest Eurasian rodent with an ecology heavily reliant on riparian woodland ecosystems^7^. It is considered a keystone species and ecosystem engineer, capable of providing multiple wetland ecosystem services and promoting biodiversity^8^. *Castor fiber* was once widespread and abundant throughout Eurasia^9^ but over-hunting and habitat loss led to severe demographic reductions and the extirpation of the species from vast areas of its former range^10,11^. By the start of the 20^th^ century, the species was on the brink of extinction with the total Eurasian population estimated to contain only 1,200 individuals^11,12^. Remaining individuals were distributed between eight scattered and highly isolated relict populations (Fig. 1), although recent evidence suggests more may have persisted^13,14^.In Britain, these declines were catastrophic. By the 16^th^ century, beavers had been entirely extirpated from this region^15^. No official attempts at reintroduction were carried out until 2009 when *C. fiber* became the first extirpated mammal to officially be reintroduced into the UK under license^16^. Eleven individuals (three families), sourced from Telemark, Norway, were released into freshwater lochs in Argyll as part of the Scottish Beaver Trial^16^. Unlicensed releases and/or escapes have also occurred along the River Tay, Scotland^17^, (with genetic origins from an admixed population in Bavaria, Germany^16,17^), and along the River Otter in East Devon, England (from an unknown origin)^18^. Additionally, in 2011, the Enclosed Beaver Project was initiated, which saw a mating pair, of unknown/unpublished origin, released into a captive site in Western Devon^19^. The success of these populations, combined with favourable feasibility studies^20,21^, indicate that large-scale reintroductions to Britain may be initiated soon.

Selection of founding members for a British beaver population should be carried out with great care. Britain was physically separated from the European landmass by sea level rise around 8.2 kya^22^. Therefore, no natural recolonisation, admixture of populations and/or external gene flow can occur as on mainland Europe. Determining the most appropriate source population/s for reintroduction is, however, confounded by the complex demographic history of the species. Relict European populations passed through severe bottlenecks of between 30 and 300 individuals^11,23^. These populations are genetically impoverished with low within-population genetic diversity, some monomorphic populations and strong phylogeographic structuring^14,24,25^. Recent evidence from ancient DNA has uncovered a huge decrease in genetic diversity within *C. fiber*, which is wholly attributed to anthropogenic influences and which has created the artificial pattern of strong population structure observed in remnant extant populations^26^. A lineage split exists for two clades, representing Western and Eastern Europe, with contact zones in Eastern Europe and NW Scandinavia^13,24,26^. Durka *et al*.^24^ proposed these lineages as Evolutionary Significant Units (ESUs) and recommended that they not be mixed. The divergence of these most likely occurred during isolation in refugia during the Last Glacial Maximum (LGM: *c*. 26,500–19,000 cal BP^27^) after which re-emergent populations experienced mixing of divergent haplotypes via contact zones.

Selection of source populations generally follows one of two broad strategies^1^, *i*) the *pre-existing adaptation strategy* or, *ii*) the *adaptive potential strategy*. In the former, population/s that bear either the closest phylogenetic similarity to the extirpated population *and/or* populations that survive under similar environmental conditions are selected. In the latter, individuals are selected from population/s which show high levels of heritable genetic variation that maximizes their potential to adapt to new environments. Any British beaver reintroduction program must, therefore, consider the ancestral relationship of the source population/s to the original British population in addition to factors such as, *i*) the relative merits of selecting founders from single versus multiple populations, *ii*) mixing individuals from disparate clades, *iii*) levels of pre-existing genetic diversity in the source population/s and *iv*) the potential for inbreeding or outbreeding depression. Reintroduction programs often aim to restore species to some, presumably arbitrary, historic baseline. However, species are not static through time and respond to a suite of biotic and abiotic influences^28,29^. Baselines are, therefore, constantly shifting and the use of heterochronous ancient DNA data permits the construction of evolutionary frameworks with the spatio-temporal complexity to reflect this. Significantly, ancient DNA allows the sampling of populations that have become regionally extinct and thus cannot be included in studies that utilize DNA from extant populations alone^30,31^ as is the case with British populations of *C. fiber*.

In this study, we employ ancient DNA analysis and ^14^C radiocarbon dating to the ancient British beaver population, for the first time, with an aim to inform conservation decision making on the appropriate source population/s for the reintroduction of beavers to Britain. We address the following questions: *i*) what is the phylogenetic relationship between ancient British beaver populations to modern and ancient European populations? And, *ii*) what was the mode and timing of post-LGM population expansion?

## Results

### Accelerator Mass Spectrometry (AMS) radiocarbon dating age estimates of *C. fiber* samples

Five samples in total were submitted to the Oxford Radiocarbon Accelerator Unit, all of which returned successful radiocarbon dates (see Supplementary Table S1, Fig. S1). One sample from Gough’s Cave, MM005 (12,386-11,836 cal BP) dated to the Younger Dryas (*c*. 12,900–11,700 cal BP), a brief cold phase of the Terminal Pleistocene immediately prior to Holocene warming. The second specimen from Gough’s Cave, MM004 (11,989-11,405 cal BP), also falls within the Younger Dryas range, although the range on the date extends into the earliest Holocene. All other samples dated within the range of the mid – late Holocene. The new dates are significant because they imply that beavers survived a period of cold-climate conditions in Britain without retreating to more temperate regions in the south. There was a small amount of overlap in the ^14^C probability distribution for MM004 and MM005 and these samples came from different skeletal elements, raising the possibility they originate from one individual. A chi-square test showed that these are significantly different from each other based on the probability distributions of ^14^C evidence (*d.f* = 1, T = 8.0, *p* < 0.05). While this is not conclusive, it does strongly suggest that the specimens are from two different individuals.

### Phylogenetic placement of ancient British beavers

A total of 17 samples successfully generated partial (492 bp) tRNA-Pro - CR sequences, and these were all located in the SW and SE of England (Fig. 1; see Supplementary Table S2). We were not able to retrieve sequences from Scottish samples. Ten haplotypes were unique. No haplotypes were shared between ancient British and modern European samples. The addition of the British data increased pairwise genetic distances, measured by F*st*, between the Western and Eastern clades from 0.61 in Horn *et al*.^26^ to 0.7 in this study. Genetic distances between the South Scandinavian sub-clade and the rest of the Western lineage were very small at F*st* = 0.054 but may have been affected by the small sample size in the South Scandinavian lineage (*n* = 6). Levels of genetic divergence between the British data and the rest of the Western clade were F*st* = 0.122, which suggests a moderate amount of genetic differentiation, despite the absence of marked phylogenetic distinctiveness.

**Figure 2:**
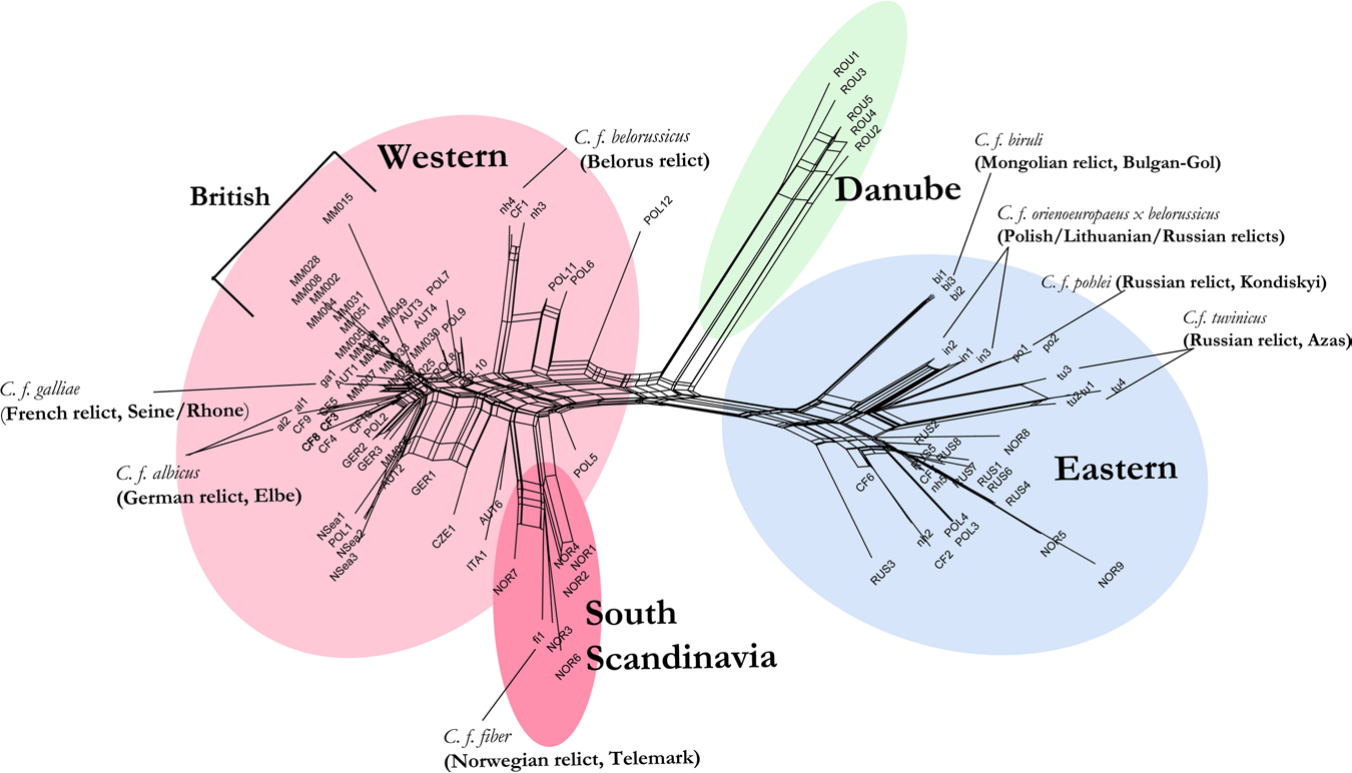
Neighbour-net network showing relationships among ancient British and modern and ancient beavers. Data is comprised of partial (492 bp) tRNAPro-Control regions mitochondrial haplotypes and comprise haplotypes generated here (prefixed ‘MM’), ancient haplotypes generated by Horn *et al*.^26,48^ and modern relict population haplotypes previously described by Ducroz *et al*.^23^, Durka *et al*.^24^ and Senn *et al*.^14^. British data are clustered closely together within the Western ESU and show no level of genetic distinctiveness from other Western populations.

**Figure 3:**
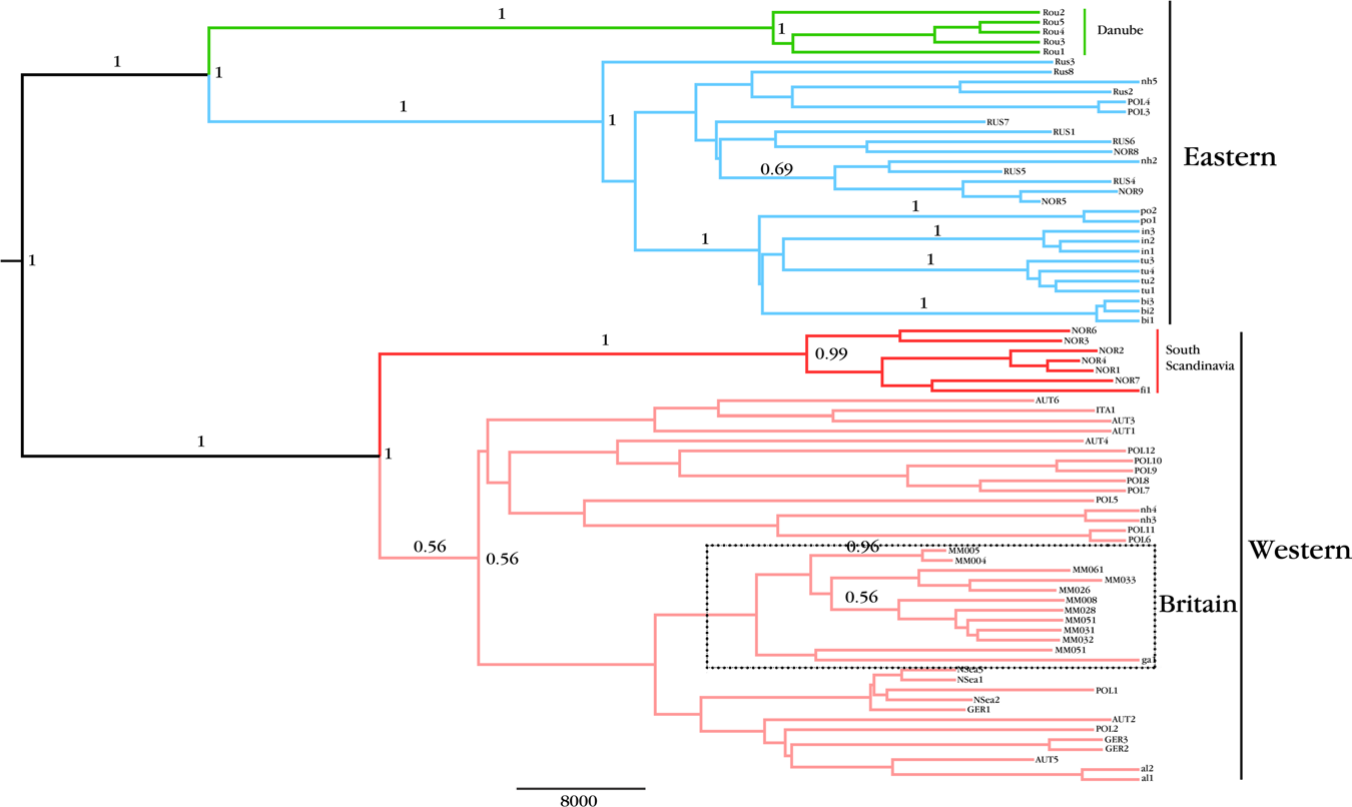
Phylogenetic relationships among ancient British and modern and ancient beavers. Shown is a maximum clade credibility (MCC) tree, generated in BEAST^47^ with partial (492 bp) tRNA-Pro/control region haplotypes, with British samples highlighted in the dashed box. Only node and branch posterior probability values above 0.5 are shown. The genealogy was generated using a Strict Molecular Clock with a fixed rate of nucleotide substitution set to 5.0 × 10^−7^ and a coalescent expansion model of demography. The tree shows the British samples group within the large Western clade with no posterior support for genetic distinctiveness.

The patterns of lineage division and placement of the British *C. fiber* haplotypes were highly congruent in both the network (Fig. 2) and phylogenetic tree analyses (Fig. 3). These recovered the topology previously reported in Horn *et al*.^26^, with a large Western clade containing a Scandinavian subclade and a large Eastern clade with an extinct subclade representing the Danube Basin. The Neighbour-Net network (Fig. 2) shows that the British data typically exhibit short to mid-length branches, cluster closely together and are nested in a large group containing other individuals belonging to the Western clade. This indicates a complete lack of substructure between British beavers and the Western phylogroup, excluding South Scandinavia, which appears moderately divergent. This suggests that the phylogenetic relationships between the ancient British data and the rest of the Western clade are highly unresolved.

The topology of the BEAST MCC tree (Fig. 3) was highly congruent with the topology returned in the network. Bayes Factors preferentially supported the scenario of a coalescent exponential growth model of demography and a clock rate of 5 × 10^−7^. However, it should be noted that there were no differences in either tree topology or support values between alternate demographic scenarios. The British data all grouped together with relict French haplotype *ga1* but there was no posterior support for the British data forming a distinct sub-lineage. Overall, there was a general pattern of unresolved phylogenetic division within the Western clade. The unresolved placement of the British data within the Western clade meant that obtaining an estimated divergence date for the British population was not possible. A range of 32, 581 – 63, 936 cal BP was returned for the time to most recent common ancestor (tMRCA) of the western clade (mean = 47,529 cal BP).

### Postglacial demography of British beavers

Tajima’s *D* statistic and Fu’s *F* returned negative and significant values for the Western clade (*D* = −1.66, *p* *=* *<* 0.05; *F* = −17.41, *p* = < 0.01) and R2 returned a significant and positive value (R2 = 0.043, *p* *=* *<* 0.05). A mismatch distribution, using a generalised least-squares approach and based on the sudden demographic expansion model, found that this clade fitted a unimodal pattern of demographic expansion (*i.e*. no significant deviation from this model was observed; sum of squared deviations (SSD) *p* = 0.96 (see Supplementary Fig. 2)). The confidence intervals for the node age estimates on the BEAST phylogeny are too large to allow a narrow age estimate for the timing of population expansion in the Western clade. However, a pre-LGM tMRCA for the Western clade, together with highly congruent tests for sudden demographic expansion, are strongly indicative of a glacial bottleneck followed by a rapid, post-LGM expansion.

## Discussion

British samples did not show any phylogenetic affinity with Eastern ESU haplotypes, therefore there was almost certainly no Postglacial recolonisation of Britain from the east. We cannot discount an Eastern European origin for earlier, Lateglacial Interstadial (*c*. 14,700 – 12,900 cal BP) populations, such as has been reported for *Arvicola terrestris*^32^, as our dataset did not contain any *C. fiber* specimens of Lateglacial Interstadial age. However, even under a scenario where Eastern European beavers contributed to Lateglacial Interstadial recolonisation of Britain, these populations are likely to have been wholly replaced by a second of wave of Early Holocene recolonisation from Western Europe. Therefore, the geographical source area for the Holocene British population was unquestionably Western Europe.

The British data all grouped unambiguously within the Western clade and showed no supported spatio-temporal substructure, either internally within British populations or between the British haplotypes and the continental Western European data. The two Late Pleistocene (Younger Dryas cold phase, *c*. 12,900 to 11,700 cal BP) specimens, MM004 and MM005, did not show any notable degree of divergence from Holocene haplotypes, suggesting that this population was contiguous with later populations and possibly contributed genetically to the establishment of these. Large polytomies in a phylogeny can be interpreted as in indicator of a rapid population expansion. This conclusion is supported here by the population expansion tests, which all strongly supported a pattern of exponential population growth for the Western clade with a tMRCA mean age of 47,529 cal BP. Given the evidence presented, we conclude that the ancient British beaver population formed part of a large, continuous, pan-Western European population. This population likely expanded rapidly from glacial refugia after the LGM, had high levels of gene flow and low levels of regional divergence within the tRNAPro-control region loci. Moreover, out of 17 haplotypes described here, 10 were unique and none were shared with extant relict populations. This adds further support to the conclusions drawn by Horn *et al*.^26^ that ancient beaver populations harboured much greater levels of genetic diversity than are seen today in post-bottleneck populations.

In the early Holocene, the coastline of NW Europe extended from NW France to Britain and, in the east, the terrestrial landscape extended from the east coast of Britain to Scandinavia, where a landbridge allowed fauna access from modern-day Denmark to Sweden^33,34^. Therefore, a huge area of suitable habitat was potentially available for woodland mammals, with few geographical barriers to gene flow in a semi-aquatic species such as the beaver. Notably, geophysical changes brought about the inundation of the British and Scandinavian land-bridges within 1000 years of each other. The Storegga Slide tsunami is hypothesised to have caused a catastrophic flood that completely severed the remaining land links between Britain and Europe *c*. 8,200 cal BP^22^. Processes associated with deglaciation of the Fennoscandian ice sheet initiated the formation of the Baltic Sea and Dana River *c*. 9,200 cal BP, which fully separated the Scandinavian Peninsula from mainland Europe^33^. Beavers likely colonized Scandinavia both from the south and east, as a contact zone can be observed in Northern Sweden and Norway^26^ (Fig. 1). South Scandinavian beavers have since become moderately divergent from the rest of the Western Europe clade whereas British beavers have not, despite spending a similar amount of time in isolation from populations on the European mainland. The reasons for this are unclear but could involve genetic factors such as founder effects and adaptation to new environments. Evidence from linear-distance metrics on ancient beaver crania has suggested that Scandinavia may have been the origin for the authochonous Scottish beaver population^35^. As we were unable to successfully retrieve full-length sequences from ancient Scottish beaver specimens, we were unable to establish the genetic relationship between the Scottish population and Southern Scandinavian beavers. However, while a distinct Scandinavian sub-clade is highly supported, the genetic distances between this group and the rest of the Western clade were extremely small.

The IUCN guidelines^3^ indicate that identifying populations that show ‘phylogenetic closeness’ to the original extirpated population is one of the key criteria when sourcing founding members for translocations. This is based on the hypothesis that these populations have adaptations already in place that will increase the likelihood of obtaining a successful breeding population in the target restoration environment^4,36,37^. However, closely related populations may not necessarily show the same genome-wide adaptive traits, particularly if they inhabit different environmental conditions, and distantly related populations may evolve similar adaptive traits via parallel evolution if they inhabit comparable environments^6^. There has been much debate in the literature as to whether phylogenetic affinity should be a consideration with regards to the British reintroductions^14,38,39^, with some authors arguing that the level of genetic adaptive potential should have higher priority^14,25^.

Halley^38^ set out three alternative scenarios for deciding on the choice of source population/s, *i*) use of the geographically closest, extant, beaver relict population, *ii*) mixture of individuals from two or more Western populations or, *iii*) release of individuals of multiple origins, regardless of ESU assignment. The lack of strong phylogeographic structure observed in this study within the Western European clade, and the low-moderate F*st* value between ancient British beavers and the Western clade, suggests that ancient British beavers did not possess significant phylogenetic distinctiveness from mainland European ancient beavers (Figs 2,3). Nor did they show any difference in the degree of phylogenetic relatedness to any of the mainland Western extant relict populations in Germany (*al* haplotypes) or France (*ga* haplotypes). Given the extremely low levels of divergence in mitochondrial control-region DNA among extant Western Europe populations, all extant Western populations show similar levels of genetic similarity to the ancient British population. Evidence from Senn *et al*.^14^ highlighted that the partition between Western and Eastern ESUs is not as well-defined as originally thought and argued that this division should be abandoned. Pending the adoption or rejection of this recommendation, the fact that the British ancient beaver population does not show close phylogenetic affinity with the Eastern clade leads us to recommend that scenarios *i*) or *ii*) from Halley^38^ should be adopted.

It should be kept in mind that there are other genetic considerations involved in choosing source populations for translocation purposes that may be given more weight than phylogenetic history. Populations of *C. fiber* are already present and persisting in Britain^17,40^. The Tay beaver population has largely been allowed to expand naturally with minimum intervention thus far. From an unknown founder number, the population now stands at around 38 family groups (*c*. 146 individuals) that appear to be thriving^17^. Genetic screening has established that these individuals descend from the Bavarian region in Germany^41^, a highly admixed population with individuals of Scandinavian, Russian and French origin^25^, *i.e* not only of mixed Western European ancestry but also originating from different ESUs. European beaver populations with admixed ancestry generally tend to have elevated levels of genetic diversity relative to unmixed relict populations and display no obvious signs of outbreeding depression^14,25^. Therefore, it may be advantageous for future beaver reintroduction projects to consider selecting founder stock that maximise genetic diversity and levels of adaptive potential above achieving close phylogenetic relatedness to the historical British population. The results presented here suggest that this would be the most appropriate path to follow given current information. Future studies may benefit from the inclusion of ancient nuclear DNA. However, as beavers do not show strong sex-biased patterns of dispersal, live in family groups and are purported to maintain a monogamous mating strategy^42^, we would expect mitochondrial and nuclear DNA to show broad phylogenetic congruence.

## Methods

### Data collection of ancient beaver bone samples

We sourced 40 bone samples of putative Terminal Pleistocene (c. 126,000 cal BP onwards) and early-late Holocene (commencing c. 11,700 cal BP) age from museum collections to represent a range of sites and chronological periods across the UK, including Scotland. We targeted dense and compact parts of the bone for both radiocarbon and aDNA sampling.

### AMS ^14^C radiocarbon dating of beaver samples

We attempted to obtain age estimates for all material that returned full-length DNA sequences. However, we could only obtain curatorial sampling permission and sufficient quantity of bone to submit five samples for AMS ^14^C dating. All sampling was done *in situ* with a hand-held drill, on sterilised surfaces. These were: MM004 (Gough’s Cave), MM005 (Gough’s Cave), MM033 (Cambridgeshire Fens), MM015 (Allermoor) and MM061 (Cambridgeshire Fens). Between 0.5 – 1 g of bone was sent to the Oxford Radiocarbon Accelerator facility (ORAU). Two of the age estimates returned for the Gough’s Cave individuals, MM004 and MM005 were very similar, which raised the possibility that they may represent a single individual. We therefore utilised the probability distributions of the ^14^C age estimates in a *chi*-square analysis to ascertain whether the two dates fall outside of each other’s 95 percent confidence ranges^43^.

### Ancient DNA extraction, amplification and sequencing

We carried out ancient DNA extractions and PCR reactions within a dedicated ancient DNA laboratory within the Natural History Museum London. All surfaces had been pre-sterilized with a bleach solution and all instruments, plastics and reagents (where appropriate) had been UV irradiated prior to use. Sampling was done with a hand-held drill with speeds below 1000 RPM. Between 10 and 50 mg of finely powdered bone was used as the basis for DNA extractions following the protocol outlined in Dabney *et al*.^44^. We targeted a partial tRNA-Pro - control region (CR) section of the mitogenome to take advantage of a large, pre-existing database of both modern and ancient sequences. We amplified the target loci in overlapping fragments with primer pairs custom designed for this study in addition to primers designed by Horn *et al*.^26^ (see Supplementary Table S3). Reactions were in 25 µl volumes per the protocol in Supplementary Protocol S1 online. Negative extractions and PCR blanks were carried out to control for contaminants. Products were purified and sequenced on an AB1 Prism 310 Sequencer. Sequences ranged in size from 81 to 130 bases in length. Base calls were manually inspected for errors then overlapping forward and reverse PCR products were aligned to a reference consensus sequence. Where low-quality peaks rendered base calling impossible, we repeated the PCR reaction and re-sequenced the products.

For 4 individuals, consistently poor-quality reads were produced. For these samples, we used Next Generation Sequencing (NGS) from DNA extracts to fill gaps. We carried out single-index DNA library builds based on the protocol by Meyer & Kircher^45^ followed by shotgun sequencing on an Illumina NextSeq500 platform. Illumina paired-end reads were merged with a mismatch cost of 2 and gap cost of 3 and were mapped to the reference consensus sequence with length and similarity fractions set at 0.94. Mapped reads were then re-aligned to a consensus sequence along with PCR generated contigs. All contigs and full-length sequences were subjected to Basic Local Alignment Search Tool (BLAST^46^) searches to rule out contamination. All sequences were translated to ensure no nonsense base calls.

### Phylogenetic placement of ancient British beavers

To search for new haplotypes in our dataset, we aligned all sequences generated in this study with all available published *C. fiber* tRNA-Pro – control region sequences. Due to the complex translocation history of beavers, many admixed populations contain individuals from mixed ESUs, which can potentially lead to inaccurate phylogenetic placement^14,25^. Therefore, when selecting modern sequences for phylogenetic analyses, we only included data from individuals known to originate from a putative relict population.

For network construction, we used all available ancient and modern tRNA-Pro and control region sequences, resulting in a database consisting of 95 sequences (see Supplementary Table S2, column F). These were aligned and used to construct a network using a neighbour-net (NN) approach. To take advantage of the available time-stamped data with which to estimate the tMRCA for the Western clade, and to infer phylogenetic placement and node ages, data that had accurate age determinations (*n* = 78) were subjected to analyses using Bayesian Evolutionary Analysis Sampling Trees^47^(BEAST). For sequences we generated in this study, all ages were determined by AMS ^14^C radiocarbon dating. In the ancient data from Horn *et al*.^26,48^, sample ages were inferred from both radiocarbon ages and associated zoo-archaeological information. All other samples included were of 21^st^ century age; Ducroz *et al*.^23^, Durka *et al*.^24^ and Senn *et al*.^14^. We included identical sequences in the BEAST run if they possessed accurate age information and the final dataset contained 78 sequences (see Supplementary Table S2). The HKY + G model of nucleotide substitution was indicated by JModelTest^49^. We investigated four clock rates, 1.0 × 10^−7^, 3.0 × 10^−7^, 5.0 × 10^−7^ and 7.0 × 10^−7^, all under the assumption of a strict molecular clock. We compared two demographic models, *i*) constant population size and, *ii*) a coalescent exponential pattern of population growth. For each separate BEAST run, we also carried out a ‘null’ simulation where sampling is performed only from the prior and not the data. Two independent MCMC chains were run for 1 × 10^8^ iterations each, sampling trees and model parameters from the posterior every 10,000 iterations. The first 10% was discarded as burn-in. Path sampling was used to estimate marginal likelihoods (MLEs) for each model. These were run on the parameters of four independent MCMC chains comprised of 100 steps of 1,000,000 generations following a burn-in of 50 percent. We then calculated Bayes Factors from the MLEs and determined model preference using the criteria of Kass & Raftery^50^. Finally, we generated a maximum clade credibility (MCC) tree, visualised using FigTree v.1.3.1^51^.

Levels of genetic divergence were estimated between, *i*) the Western (including British data, *n* = 68) and Eastern (*n* = 27) phylogroups and, *ii*) the British data (representing a regional, geographic, population, *n* = 17), and the remainder of the Western clade (*n* = 51). F*st* values, based on the methods of Hudson *et al*.^52^ were calculated in DNAsp v.5.0^53^. When applied to mitochondrial DNA these are based on differences in haplotype frequencies and assume the island model of population structure^54^.

### Postglacial demography of Western beavers

To test whether the data are consistent with a neutral model of evolution or, alternatively, show a signature of rapid population expansion, we performed three tests of neutrality for the Western clade only, with a total sample size of *n* = 68. These were Tajima’s D, Fu’s F^55^ and R2^56^. Both the Tajima’s D and Fu’s *F* values are expected to be equal to zero under a null hypothesis of population equilibrium and selective neutrality; significant negative values for Fu’s *F* and Tajima’s *D* and significantly positive values for R2 can be interpreted as a signature of population expansion. We assessed the significance levels of tests via the generation of 1,000 random samples. The Western clade was also examined for evidence of a population experiencing exponential growth by carrying out a mismatch distribution in Arlequin v 3.5^57^ based on the sudden demographic expansion model. If populations have passed through a demographic expansion, this distribution will fit a unimodal curve^58,59^.

### Data Availability Statement

The datasets generated during the current study are available in the Genbank repository https://www.ncbi.nlm.nih.gov/genbank/ under accession numbers MG773796-812.

## Electronic supplementary material

Supplementary Information 1

Dataset 1
