# Supplementary material for: Identifying source populations for the reintroduction of the Eurasian beaver, Castor fiber L. 1758, into Britain: evidence from ancient DNA
Source: Sci Rep. 2018 Feb 9;8:2708. doi: 10.1038/s41598-018-21173-8 (PMC5807398; doi:10.1038/s41598-018-21173-8)
Supplement: Supplementary file 1 — Supplementary Information 1 [file 41598_2018_21173_MOESM1_ESM.doc]

**Supplementary Information.**

**Identifying source populations for the reintroduction of the Eurasian beaver, *Castor fiber* L. 1758, into Britain: evidence from ancient DNA**Melissa, M. Marr^1,2*^., Selina Brace^2^., Danielle, C. Schreve^1^., Ian Barnes ^2^.

^1^Department of Geography, Royal Holloway University of London, Egham Hill, Egham, Surrey, TW20 0EX
^2^Department of Earth Sciences, Natural History Museum London, Cromwell Road, South Kensington, London, SW7 5BD

*Corresponding author: [melissammarr1981@gmail.com](mailto:melissammarr1981@gmail.com); Tel: +44 7724 255969

**Supplementary Table S1. AMS radiocarbon dates for all *Castor fiber* samples which generated successful tRNAPro – Control Region sequences and which were used in the phylogenetic analyses in this study.**

| **ID** | **Year Dated** | **Uncalibrated C^14^ Date** | **Error** | **Calibrated C14 date (95.4% Prob)** | **OxA ID** | **Citation** |
| --- | --- | --- | --- | --- | --- | --- |
| MM002 | 2002 | 4285 | 45 | 4973-4812 | 11,093 | Coles^33^ |
| MM004 | 2016 | 10110 | 45 | 11989-11405 | 33,525 | This Study |
| MM005 | 2016 | 10300 | 50 | 12386-11836 | 33,526 | This Study |
| MM008 | 2002 | 4150 | 40 | 4829-4569 | 11,094 | Coles^33^ |
| MM015 | 2016 | 4726 | 29 | 5583-5326 | 33,524 | This Study |
| MM026 | 2002 | 4375 | 40 | 5210-5205 | 11,090 | Coles^33^ |
| MM028 | 2002 | 4032 | 37 | 4783-4767 | 11,218 | Coles^33^ |
| MM031 | 2002 | 4265 | 40 | 4960-4807 | 11,092 | Coles^33^ |
| MM033 | 2016 | 3870 | 33 | 2466-2211 | 33,685 | This Study |
| MM051 | 2002 | 4085 | 40 | 4813-4755 | 11,088 | Coles^33^ |
| MM061 | 2016 | 3899 | 29 | 4418-4247 | 33,529 | This Study |


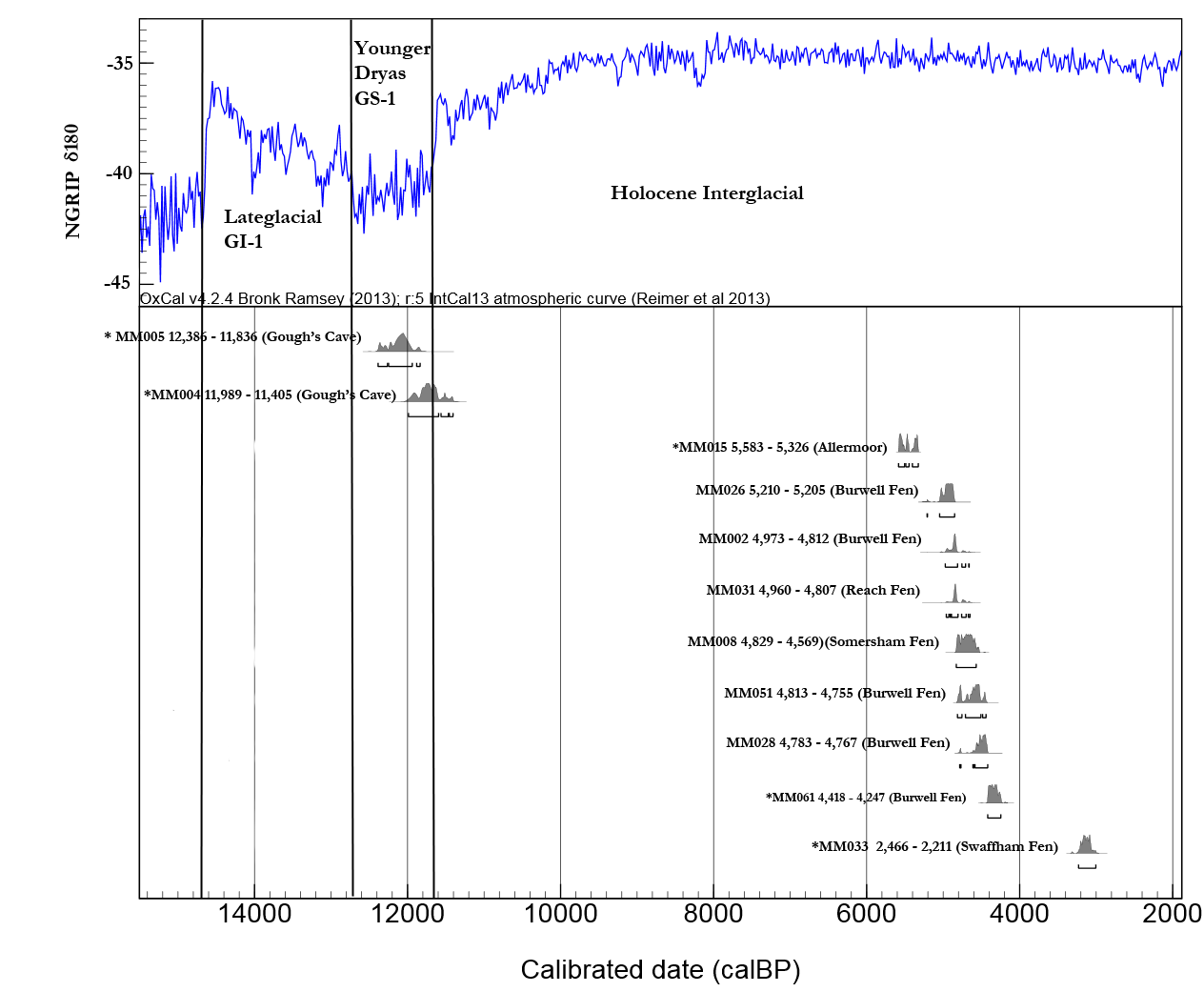


**Supplementary Figure S1.** **AMS radiocarbon dates for all *Castor fiber* samples which generated successful tRNAPro – Control Region sequences and which were used in the phylogenetic analyses in this study.** Samples dated specifically by this investigation are denoted by ‘*’. Two samples have ages which place them in the Younger Dryas cold phase while the remainder of samples date to the mid - late Holocene Interglacial.


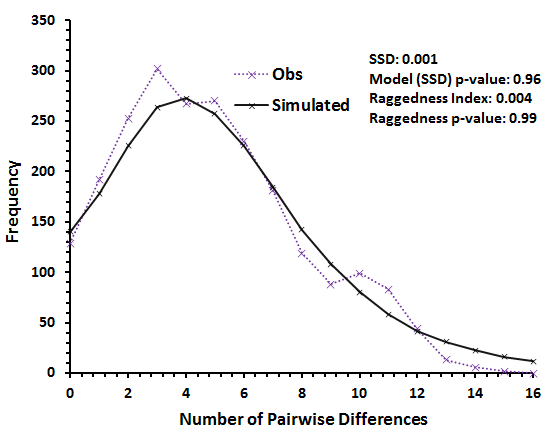


**Supplementary Figure S2.** Mismatch distribution of observed frequencies of pairwise differences for *C. fiber* within the Western European clade. Simulated data show expected frequencies under a hypothesis of population expansion; observed data show observed pairwise differences in the dataset.

**SI Table S3**. Primer pairs and their properties used in amplification of a 492bp sequence of the *Castor fiber* control region. Please note that where necessary these were supplemented by primers designed in Horn *et al.*^25^

| **Primer** | **Sequence 5' - 3'** | **Size (bp)** | **GC%** | **Tm** | **Product Size (bp)** |
| --- | --- | --- | --- | --- | --- |
| CF1_Test_F | TGGTCGTTCATTAGCGCATT | 20 | 45 | 58.3 | 99 |
| CF1_Test_R | TGGTTTCACGGAGGATGGTA | 20 | 50 | 58.4 |
| CF2_Test_F | CGAGCAGGAATAACAAGCACG | 21 | 52 | 59.9 | 89 |
| CF2_Test_R | TCCATGCTGGATTGGTGTTATGT | 23 | 43 | 60.3 |
| CF1F | ACCAGCAATGGGGAAACACT | 20 | 50 | 59.8 | 81 |
| CF1R | GAGAATGTCAGCTTTGGGTGC | 21 | 50 | 59.8 |
| CF2R | TCAGGGGAGAAACAAAGTCC | 20 | 50 | 57 | 85 |
| CF2R | TGTACATGTATATGTTACAGGGGA | 24 | 38 | 56.7 |
| CF3F | TCTCATAATTAAACTATCCCCTGT | 24 | 33 | 54.8 | 85 |
| CF3R | TGTGGAATATAACATAATGCACG | 23 | 35 | 55.2 |
| CF4F | ACAACAGTCTATGTATATCGTGCA | 24 | 38 | 57.9 | 92 |
| CF4R | ACAGTTATGTACTATGTACGATTAGCA | 27 | 33 | 58.2 |
| CF5F | GCTTGAGTACTTCAAATGCTAATCG | 25 | 40 | 58.8 | 88 |
| CF5R | ACGTGCTTGTTATTCCTGCTC | 21 | 48 | 58.9 |
| CF6F | CCCGAGCAGGAATAACAAGC | 20 | 55 | 59 | 90 |
| CF6R | CCATGCTGAATTGGTGTTATGTTG | 24 | 42 | 59.1 |
| CF7F | TAATCTATTGGTCGTTCATTAGCGC | 25 | 42 | 59.1 | 91 |
| CF7R | GCGCTAATGAACGACCAATAGA | 22 | 45 | 58.9 |
| CF8F | TAATCTATTGGTCGTTCATTAGCGC | 25 | 40 | 59.6 | 85 |
| CF8R | TCAAAAGTTTGAACTGTGGTAGGG | 24 | 42 | 59.4 |
| CF9F | GGATATCCCCTACCACAGTTCA | 22 | 50 | 58.7 | 86 |
| CF9R | AGCGAGAAGAGGGACATTCC | 20 | 55 | 58.7 |
| CF10F | GGAATGTCCCTCTTCTCGCT | 20 | 50 | 59.2 | 86 |
| CF10R | CCTGAAGTAAGAACCAGATGCC | 22 | 55 | 59.2 |
| GF1F | ACAACAGTCTATGTATATCGTGCA | 24 | 38 | 57.9 | 130 |
| GF1R | TGTTATTCCTGCTCGGGGAAC | 21 | 52 | 60.1 |
| GF2F | AGTACATAACTGTATAATCGTACA | 24 | 29 | 52.9 | 126 |
| GF2R | ATCCATGCTAGGTTGGTGTT | 20 | 40 | 55.6 |
| GF3F | CAACATAACACCATTTTGAAGA | 20 | 40 | 53.1 | 130 |
| GF3R | GGGGATATCCATTTTGAAGA | 20 | 40 | 52.2 |
| GF4F | ACAGTTCTCTTCAAAATGGA | 20 | 35 | 52.3 | 130 |
| GF4R | AGAAACCCCCAGAACGTATG | 20 | 50 | 57.9 |

**SI Protocol S1.**

**PCR Reactions.** PCR reactions were performed in 25µl volumes with final volumes of; 0.5 U of HotStart Taq DNA polymerase, 1x Taq polymerase buffer with (NH_4_)_2_SO_4_, 0.5µM MgCI_2_, 0.1mg/mL Bovine Serum Albumin (BSA), 0.2µM of deoxynucleoside triphosphates (dNTPs) and 0.4µM of each primer. Cycling conditions were: initial denaturation at 95^o^C for 5 mins followed by 50 cycles of denaturation at 94^o^C for 1 min, annealing at between 49^o^C to 51^o^C (depending on optimal annealing temperature for each primer pair) for 1 min and extension at 72^o^C for 1 min with a final extension at 72^o^C for 10 mins. Negative extraction controls and negative PCR controls (with H_2_O) were performed with each round of PCR. Amplicons were visualised on 2% agarose gels and products of successful amplifications were purified using 0.15x Ampure Beads to remove primer dimer and residual contaminants of the PCR process. Sequencing was performed commercially by Macrogen on an AB1 Prism 310 Sequencer with primers at 5pM.
